# Investigation of Pomegranate (*Punica granatum* L.) Flowers’ Antioxidant Properties and Antibacterial Activities against Different *Staphylococcus* Species Associated with Bovine Mastitis

**DOI:** 10.3390/vetsci10060394

**Published:** 2023-06-11

**Authors:** Gülten Ökmen, Daniela Giannetto, Francesco Fazio, Kutbettin Arslan

**Affiliations:** 1 Department of Biology, Faculty of Science, Mugla Sıtkı Kocman University, Mugla 48000, Turkey; gultenokmen@gmail.com (G.Ö.); danielagiannetto@gmail.com (D.G.); kutbettinarslan@gmail.com (K.A.); 2 Department of Veterinary Sciences, Messina University, 98166 Messina, Italy

**Keywords:** *Punica*, mastitis, antibacterial activity, antioxidant activity, TLC

## Abstract

**Simple Summary:**

Mastitis is known to pose a public health hazard and cause a costly disease for dairy herds. Bacteria are the primary causes of mastitis; and antibiotics are widely used in the treatment of the disease. This can lead to antibiotic residues in various kinds of milk and an increase in the risk of antibiotic resistance in bacteria. To avoid these problems, alternative treatments by the use of natural products are being experimented and plants extracts are also examined. This study reports the investigation of potential antioxidants and antibacterial activities against mastitis pathogens of pomegranate flowers’ extracts. Results showed a high antioxidant and antibacterial potential of pomegranate flowers’ extracts against the examined mastitis pathogens.

**Abstract:**

Mastitis is one of the most considerable and costly diseases for dairy herds, and *Staphylococcus* spp. is known to be the main causative agent. Although antibiotics are widely used in the treatment of mastitis, this can cause both antibiotic residues in milk and the risk of antibiotic resistance occurrence in bacteria. Thus, in recent years, researchers have focused on alternative treatments for this disease and plants extracts are investigated for this purpose. Pomegranate is widely used as a dye, ornament, and medicinal plants in the industry, and the species has a particularly high economic value in Turkey. This study aims to investigate in vitro the antioxidant and antibacterial properties of the pomegranate flower’s extracts against different *Staphylococcus* species associated with bovine mastitis. To this aim, pomegranate flowers were collected from different regions in Turkey and extracts were prepared with three different solvents (methanol, ethanol, and water). The retention factor values of the ethanol extract were determined by thin-layer chromatography. The antibacterial activity tests were carried out via the disk diffusion method. In addition, the extracts were tested against the stable DPPH (2,2-diphenyl-1-picryl-hydrazyl-hydrate) free radicals for antioxidant activity. Four retention factors (0.79, 0.67, 0.58, and 0.33 points) were found for the ethanol extract. The methanol extract showed the highest inhibition zones against coagulase-negative *Staphylococcus*-37 (CNS-37) and *S. aureus*-18. The lowest MIC was 6500 μg/mL. The highest antioxidant activity was observed in methanol extracts. As a result, the extracts of pomegranate flowers showed a high antioxidant and antibacterial potential against the examined mastitis pathogens.

## 1. Introduction

To obtain products from ruminant animals and make them sustainable, it is necessary to prevent or control possible diseases. Mastitis is one of the most important diseases affecting dairy herds, and thus, is one of the costliest for the dairy industry [1,2]. *Staphylococcus* spp. is the main causative agent of mastitis, but these bacteria are known to show multiple resistance to antimicrobials, posing a higher risk to public health [3,4,5]. *S. aureus* is one of the main pathogens isolated from bovine mastitis in different countries with a prevalence of 74% in Ethiopia [6] and Canada [7], 47.2% in herds from Italy, and 41% in France [8]. Other accountable pathogens causing mastitis are *Streptococcus agalactiae*, *Escherichia coli*, *Streptococcus uberis*, *Klebsiella* spp., *Prototheca* spp., and other organisms such as fungi, mycoplasmas, and algae [9]. The interactions between the immune system of dairy herds and the pathogen agents causing mastitis can lead to irreversible damage to the secretory part of the udder that, in the worst cases, can no longer be functionally capable to produce and secrete milk [10]. Thus, animal immune response plays a key role in the pathogenesis of mastitis as the first line of defense against the pathogen invasion of the udder.

In recent years, there has been an increase in the interest in the synergic role of stress and immunity in farmed animals [11,12]. Animals try to react to several stressors through complex mechanisms which act to maintain homeostasis in their bodies. This is very complicated and involves a series of physiological processes comprising numerous molecular and cellular defense mechanisms [13]. When oxidative stress occurs, the body produces an excessive release of reactive oxygen species (ROS) that cannot be eliminated by the normal antioxidant mechanisms [14]. Recent studies have focused on the role of external antioxidant molecules in reducing this oxidative stress and its consequent cellular damage [15,16,17].

Phytobiotics are secondary plant metabolites, such as flavonoids, steroids, alkaloids, terpenes, tannins, phenolics, and essential oils, existing in plants as normal constituents or as a result of pathogen invasion [18]. Phytobiotics are synthesized by plants as a protection mechanism against invasive pathogens and they can also act to protect DNA or the photosynthetic apparatus from oxidative damage caused by ultraviolet radiation [19]. They are also known as non-nutritive compounds in contraposition to other nutrient compounds such as vitamins and minerals found in plants. They are widely used in animal farming because they can have benefits for health and growth [18,19].

Within the previous year, the interest in the use of these metabolites as efficient antioxidants, as alternative control agents for reducing the number of resistant microorganisms and antimicrobial residues in the food of animal origin, and also in their immunomodulatory and therapeutic effects on various diseases and disorders has noticeably increased [20,21,22,23,24,25,26,27,28].

Pomegranate (*Punica granatum* L.) is a plant belonging to the Lythraceae family that consists of herbaceous annuals, or perennials, shrubs or trees. The family is mostly distributed in tropical regions and is represented by 31 genera and 600–620 species in the world [29]. Pomegranate is widely considered native throughout the region from Iran to northern India as apparently wild plants in many forests in this region, but has been naturalized throughout the Mediterranean and North Africa [29]. Pomegranate is a thorny bush or small tree 2–7 m tall with flowers that are scarlet-red, rarely white [30,31]. The edible fruits are widely used for various purposes in food, cosmetics, and tint industries [32,33]. In addition, pomegranate and its components such as seeds, leaves, barks, fruits, and juice have been reported to have various pharmacological and therapeutic properties [34,35,36,37]. Extracts of *P. granatum* peels are effective in combating different types of bacteria and may be used to control bacterial infections due to its polyphenol content [38,39,40,41,42]. With regard to *Staphylococcus* spp., different researchers have reported high antimicrobial activity against *S. aureus* of pomegranate peel [43] and bark extracts [44] and high capacity to improve biochemical and histological parameters in mice infected with *S. aureus* [45].

Although many studies in the literature have highlighted the antimicrobial and antioxidant effect of the different parts of pomegranate (seeds, bark, juice, or pericarp) [46], no specific study investigating the pomegranate flowers’ activities against mastitis pathogens has been found. Pomegranate flowers are known to contain a variety of secondary metabolites [47]. The most abundant are polyphenols, including gallic acid [48], ellagic acid, and ethyl brevifolin-carboxylate [49], that have strong antioxidant activity [50]. Thus, this study aims to investigate in vitro antioxidants and antibacterial activities against eight *Staphylococcus* bacteria associated with bovine mastitis for the extracts of *Punica granatum* flowers, collected from different regions of Turkey and prepared using different solvents.

## 2. Materials and Methods

### 2.1. Collection and Preparation of Plant Material

Pomegranate flower samples were collected from different regions in southwestern Turkey (Mugla, Denizli, and Isparta). All plant materials were identified to belong to the species *Punica granatum* according to [27]. However, in order to detect potential differences in the activities in the plants collected from different areas, the pomegranate flowers collected from different locations were treated as three different samples and separate extracts were prepared. After collection of the samples, they were transported to the Laboratory of Microbial Biotechnology at Muğla Sıtkı Koçman University (Turkey). Then, they were washed 2–3 times under running water and once in sterilized and distilled water. The plant samples were then air-dried and ground into a powder with a blender. All materials were stored at room temperature until sample preparation, then stored at 4 °C until extraction.

### 2.2. Preparation of Plant Extracts

Three different solvents were used in this study: methanol, ethanol, and water at 250 mL. These three solvents were chosen for their similar polarity to extract the polar natural contents of the plants’ materials (such as polyphenols and flavonoids) and for their lower toxicity when compared with other potential solvents (such as chloroform and dichloromethane). Air-dried and powdered samples were extracted using these three solvents by using a Soxhlet apparatus. Firstly, the obtained dry material was pulverized into a powder using a blender (Fakir, Vaihingen an der Enz, Germany). After pulverization, the samples were preserved in the dark at 4 °C until extraction. Then, 50 g of the pulverized samples were weighed and placed into a Soxhlet apparatus (Isotex, Ankara, Turkey) for extraction using methanol, ethanol, and aqueous solvents (250 mL) for 4 to 8 h. After the extracts in organic solvents were evaporated, each of them was stored in its own solvent in small sterile opaque bottles at 4 °C until further examination. The percentage yield of different plant extracts with different solvents were: water, 19%; ethanol, 24.2%; and methanol, 22.6%.

### 2.3. Thin Layer Chromatography

Thin-layer chromatography (TLC) was applied to examine the components of the plant extract, and retention factor (Rf) values were determined. To this aim, the components of *P. granatum* ethanol extract were examined. TLC silica gel 60 F 254 aluminum plates (Merck, Frankfurt, Germany) were cut into 10 × 10 cm sizes. A 1 cm gap was left from the edges and bottom of the plates. In the study, chloroform/methanol solvents were used in a ratio of 7:3, respectively. The sampling was made with capillaries in the form of 5 spots and the spots were dried by a dryer. The plate was placed in the solvent tank obliquely so that 0.5 cm from the bottom remained in the solvent mixture. The run was continued for about 15 min and then the plates were left to dry at room temperature, after which, the Rf values were determined [51,52]. Rf values were calculated using the formula Rf = x1/x0 (x1 = distance traveled by the solute, x0 = distance traveled by the solvent).

### 2.4. Preparation of Bacteria Material

Eight bacteria that are well-known to be responsible for bovine mastitis were used in this study: *Staphylococcus aureus*-17, *Staphylococcus aureus*-18, and 6 coagulase-negative (CNS) *Staphylococcus* spp. (CNS-22, CNS-29, CNS-32, CNS-33, CNS-36, and CNS-37). All the bacteria materials were obtained from previous studies of the author (G.Ö) and were stored in the Laboratory of Microbial Biotechnology. For the purposes of this study, the stored bacterial cultures were grown in Mueller–Hinton Broth (Merck, Frankfurt, Germany) medium at 37 °C for 24 h.

### 2.5. Determination of In Vitro Antibacterial Activity and Minimum Inhibitory Concentration (MIC)

Antimicrobial activity tests were performed according to [53]. The plant extracts (300 mg/mL) were tested against bacteria by using the disk diffusion method. Accordingly, the turbidity of bacterial cultures was set to 0.5 McFarland, and 0.1 mL was inoculated onto the plates under aseptic conditions. Then, empty disks (6 mm) (Bioanalyse, Ankara, Turkey) were soaked with 25 μL plant extracts and placed on the plate surface. The cultures were incubated on Mueller–Hinton agar plates (MHA, Merck, Frankfurt, Germany) at 37 °C for 24 h. After incubation, the formed inhibition zones were recorded in mm. Oxacillin (5 µg) was used as the standard antibiotic (control) in this study.

The minimum inhibitory concentrations (MIC) of pomegranate flower extracts were also applied to further test the antibacterial activity. MIC was considered as the lowest concentration that inhibited growth after incubation. The broth dilution method was applied as defined in the CLSI standards (CLSI, 2003; CLSI, 2006). This test was adjusted to the final concentrations of each extract 13,000, 6500, 3250, 1625, and 812.5 µg/mL.

### 2.6. Determination of Non-Enzymatic Antioxidant Activity

DPPH (2,2-diphenyl-1-picryl-hydrazyl-hydrate) was used to determine the free radical scavenging activity of the pomegranate flower extracts. This method is known to be the most suitable procedure for determining antioxidant activity [43]. The 0.1 mL of extract was added to 2.9 mL of methanol DPPH solution (0.1 mM). After 30 min of incubation, the absorbance of the extract was measured at 515 nm using a spectrophotometer. DPPH solution with methanol was used as control, and methanol was used as blank. Trolox was used as the reference antioxidant. The results were calculated using the DPPH scavenging capacity formula and given in percentage [54].

## 3. Results

**Figure 1:**
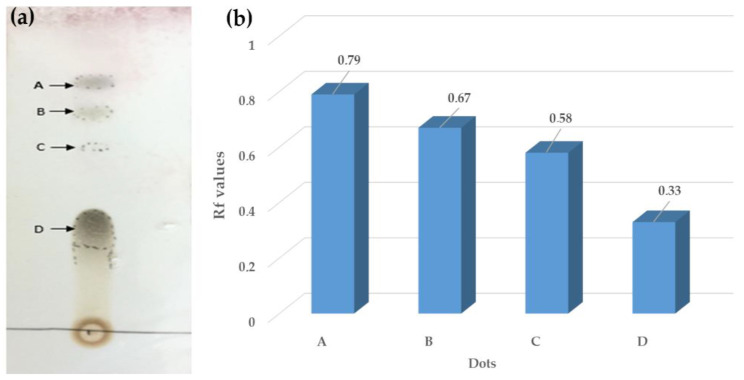
(**a**) TLC image of *Punica* flowers’ ethanol extract and (**b**) Rf values of *Punica* flowers’ ethanol extracts.

The presence of various components of pomegranate flowers (ethanol extract) was confirmed by thin-layer chromatography (TLC). The TLC profile is reported in Figure 1a. Four Rf values (0.79, 0.67, 0.58, and 0.33 points) were specified for the pomegranate ethanol extract (Figure 1b). This result revealed the presence of phenolic compounds of four different polarities and only one of the spots was found below 0.5, whereas the rest of the spots were above this value. The TLC profiling results suggested a highly active phytochemical presence within chloroform/methanol (7:3) crude extract for *Punica granatum* flowers’ ethanol extract.

**Figure 2:**
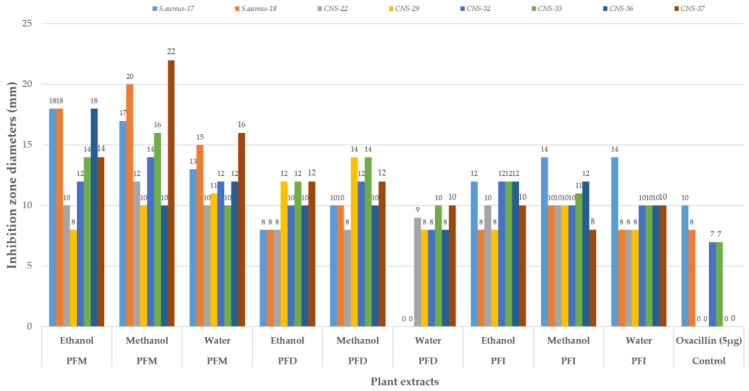
Antibacterial activities of *Punica granatum* flower extracts collected from different provinces; PFM: *Punica* flowers of Mugla; PFD: *Punica* flowers of Denizli; PFI: *Punica* flowers of Isparta; *S. aureus*: *Staphylococcus aureus*; CNS: coagulase-negative *Staphylococcus*; ethanol: ethanol extract; methanol: methanol extract; water: water extract.

The results of the in vitro antibacterial activity of pomegranate flowers’ extracts tested against the mastitis microorganisms are given in Figure 2. All results are presented in diameters of the inhibition zone (in mm). Considering the samples collected in different regions as separated, all the pomegranate flower extracts (except for the water extract of samples collected from Denizli) showed a high activity against mastitis pathogens. More precisely, the highest antibacterial activities were observed in the pomegranate flower extracts collected from Mugla: the methanol extract (22 mm and 20 mm zone diameters against CNS-37 and *S. aureus*-18) followed by the ethanol extract (18 mm zone diameters against *S. aureus*-17, *S. aureus*-18 and CNS-36) and then the others (Figure 2). 

When the effects of standard antibiotics and standard solvents on the bacteria were analyzed, none of the standard solvents showed any activity against the examined bacteria, whereas the standard antibiotic showed its zones only against *S. aureus*-17 (10 mm), *S. aureus*-18 (8 mm), CNS-32 (7 mm), and CNS-33 (7 mm). 

The results of the minimum inhibitory concentration are shown in Table 1. The MIC values were found to be high, especially in the extracts prepared with pomegranate flowers collected from Mugla, but also for all the samples collected from the other two regions. 

**Figure 3:**
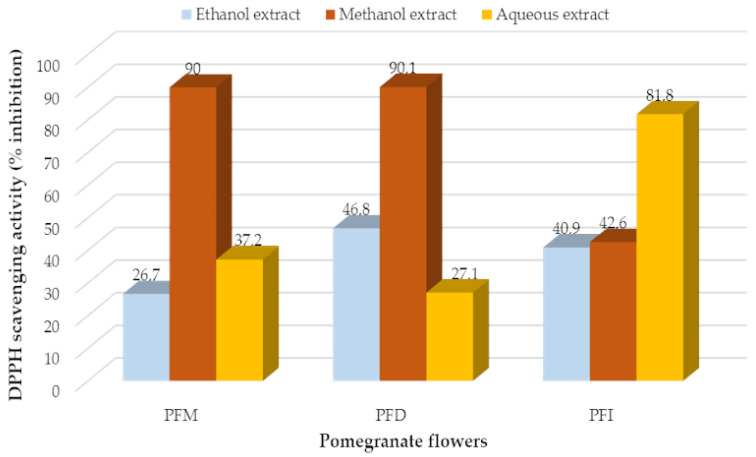
DPPH radical scavenging activities of different extracts of pomegranate flowers. (PFM: *Punica* flowers of Mugla; PFD: *Punica* flowers of Denizli; PFI: *Punica* flowers of Isparta).

Considering the results of the DPPH, the highest antioxidant activity was determined from the methanol extract of Denizli pomegranate flowers (90.1%), followed by the methanol extract of Mugla (90.1%) and the water extract from Isparta (81.8%) (Figure 2). The lowest antioxidant activity was observed from the ethanol extract of Mugla pomegranate flowers (26.7%) and the water extract of Denizli flowers (27.1%) (Figure 3).

## 4. Discussion

The excessive use of chemical synthetic drugs causes increased resistance in microorganisms, and hypersensitivity, immune suppression, and allergic reactions in animals and also in humans [55]. Therefore, there is a need for natural alternative compounds that can be used as antioxidants or antibacterial agents [56]. Accordingly, there is an increasing demand toward the use of herbal medicines and their active ingredients [57,58,59,60]. The advantages of plant-derived compounds are that they usually have no side effects, are affordable, and have therapeutic potential for the cure of many diseases [61,62]. For these reasons, the screening of plants continues with the hope of discovering pharmacological antimicrobial tools that are very effective and safe for antimicrobial assays.

Previous studies in the literature reported antimicrobial activities of different plants’ extracts against *S. aureus* mastitis pathogens: *Elaeagnus angustifolia* extracts [63] and *Liquidambar orientalis* [64] showed antibacterial activities against *S. aureus*-17, whereas [65] reported antibacterial activities of *Piper nigrum* extracts and *Ocimum basilicum* extracts [66] against *S. aureus*-18.

Considering the composition of the examined extracts, four Rf values were found for the pomegranate flowers’ ethanol extract. The authors of [67] applied the toluene/ethyl acetate/formic acid system for the fruit peel of pomegranate, and they found a single zone with an Rf value of 0.9. The authors of [68] reported six spots at three different wavelengths with Rf values between 0.20 and 0.92 for the extract derived from pomegranate peel. The authors of [69] described eleven spots with Rf values between 0.14 and 0.80 for pomegranate alcohol extract. Finally, the authors of [70] reported some phenolic compounds in the juice of the pomegranate fruit and the phytochemical analyses reported the presence of caffeic and chlorogenic acids in the extracts.

The antimicrobial properties of *P. granatum* have been explored in recent years [59,71,72,73]. The ethanol, water, methanol, and acetone extracts of *P. granatum* showed potent antimicrobial properties against Gram-positive and Gram-negative non-oral microorganisms [74,75]. Furthermore, several studies evaluated the antibacterial effects of this herb on oral bacteria [76,77,78].

In the current study, all the pomegranate flower extracts (except for the water extract of samples collected from Denizli) showed high activity against mastitis pathogens (22 mm and 20 mm zone diameters against CNS-37 and *S. aureus*-18 for the methanol extract and 18 mm zone diameters against *S. aureus*-17, *S. aureus*-18, and CNS-36 for the ethanol extract). 

A previous study investigating methanol, ethanol, ethyl acetate, and acetone extracts of pomegranate fruit peels against 10 different bacteria, including *S. epidermidis* ATCC12228, *S.aureus* ATCC29213, reported inhibition zone diameters between 18–30 mm [79]. Similarly, another research investigating the antibacterial activities of aqueous and methanolic extracts of pomegranate leaves against bovine mastitis pathogens reported that methanolic extracts showed the lowest (25 mm) and the highest (32 mm) zones, and for the aqueous extract, 25–36 mm against these pathogens [80]. In a study on the bioactive compounds of aqueous and methanolic extracts of the pomegranate plant against bovine mastitis source pathogens, it was found that the compounds of the methanol extract presented 12 mm and 16 mm inhibition zones against *S. aureus* and coagulase-negative *S. aureus* [81]. Pomegranate fruit extracts resulted in a 13–39 mm inhibition zone [82], whereas aqueous and ethanol extracts resulted in a zone of 25.5 mm against *S. aureus* [44]. Similarly, an aqueous fraction of pomegranate (500 mg/mL) presented an inhibition zone of 16–32 mm against *S. aureus* ATCC 25,923 [75]. Two studies testing the pomegranate peel extract against *S. aureus* found zone values between 22–24 mm [83] and 12–22 mm [84].

All these studies had similar results with the current study, which suggested that the antimicrobial activities of the pomegranate flower were as high as in those of other parts of the plant. Although in the current study, all the extracts showed high antimicrobial activities, the extract of pomegranate flowers collected from different regions of Turkey revealed slightly different results. These differences, similar to those between the results of the aforementioned studies, could be attributed to the composition of the bark, environmental factors, or postharvest of the plant [85]. 

The antimicrobial activities of *Punica granatum* extracts might be related to the presence of phenolic compounds that may involve multiple mechanisms of action. For example, it may diminish the cell wall, interact with its composition, and disrupt the cytoplasmic membrane [86], degrade membrane protein, interfere with membrane-integrated enzymes [87], alter fatty acid and phospholipid components, disrupt enzymatic mechanisms for energy production and metabolism, modify nutrient uptake and electron transport [88], affect the synthesis of DNA and RNA, and destroy protein translocation and the function of mitochondria in eukaryotes [89]. In addition, the pomegranate was reported to contain oligomeric ellagitannin, which is the most potent antibacterial compound in this plant and, together with other compounds such anthocyanins (pelargonidin-3-galactose and cyanidin-3-glucose) and flavanols (quercetin and myricetin), it could act synergistically to contribute to the antibacterial effect of pomegranate [90].

The most important factors affecting MIC were the different compositions of the extracts, the geographical location of the plant, the harvest season, the age of the plant, the growth stage, the drying method, and the extraction technique, respectively [91]. The value obtained in this study was higher than that of [92], which reported an MIC value of 0.19 mg/mL against *S. aureus*.

The ability of *S. aureus* to cause infections were associated with the expression of various virulence factors. One of these was the capacity of some *S. aureus* phenotypes to produce biofilms that might be related to antimicrobial resistance [93,94]. The genes inducing biofilm formation and their exact roles are still not well-known and warrant more attention. In this regard, the present study has some limitations as it examined only a limited number of genes and did not focus on their potential association with the biofilm phenotype. In addition, given that in vitro observations of biofilm formation were not easily comparable with the in vivo events occurring in the udder of dairy cows with mastitis [95,96], future in vitro and in vivo studies focusing on more genes and their association with biofilm formation in mastitis are strongly suggested.

With regard to the antioxidant properties, the potent antioxidant activities of pomegranate were attributed to its high content of polyphenols, including ellagic acid in its free and bound forms, gallotannins and anthocyanins (cyanidin, delphinidin and pelargonidin glycosides), and other flavonoids (quercetin, kaempferol and luteolin glycosides) [97,98]. Among these polyphenols, the punicalagin was reported to be the bioactive constituent responsible for 50% of the pomegranate fruit juice’s antioxidant activity [98,99]. 

The results of the test on antioxidant activities in this study using the pomegranate flowers’ extracts showed higher antioxidant activities compared with the results reported in previous studies using other parts of pomegranate plant. The authors of [100] examined the antioxidant activity of the pomegranate collected from four different regions in Turkey (Hatay, Hicaz, Adana, and Antalya) and reported an EC50: 150.7 mg/L for pomegranate collected from Antalya and EC50: 74.1 mg/L for the samples collected from Hatay. The authors of [101] studied the DPPH IC50 values of water and ethyl acetate extracts of pomegranate seeds, fruits, and peels and reported an IC50 value of 15,100 mg dry weight/mL for the pomegranate seed ethyl acetate extract. The authors of [102] investigated the pomegranate fruits’ extracts and observed an antioxidant activity of 64.9%.

Despite these findings, it is important also to consider that the effects of the extracts might vary by the content variation and stability during storage [103]. The lack of tests for analyzing the stability during the examination periods of the extracts is another limitation of the study. Further studies focusing on the analysis of the stability during the experimentation of these extracts are also highly suggested. 

## 5. Conclusions

Mastitis is one of the major problems in the dairy industry that causes a decrease in yield. This may lead to serious economic losses as a result of the increased drug use, veterinary expenditure, and the exclusion of animals from breeding. The maintenance and modulation of redox homeostasis in livestock is thus essential for animal welfare and to guarantee the best quality of the derived products. Therefore, it is suggested that natural agents, which are able to act against the bacteria and to reduce the oxidative stress occurring in cells after infection, can be used in appropriate doses. Plants’ extracts such as those of the pomegranate may be the best samples for these studies due to their common usage and easy access. The high antibacterial and antioxidant activity of the pomegranate flower extracts against mastitis pathogens observed in this study reveal that the flowers, similar to other parts of the plants, are an appropriate candidate to be used for the further development of medication, which can be used against the pathogens responsible for mastitis and also to reduce the oxidative stress occurring after infection. Future phytochemical studies focusing on the determination of the exact components as well as in vivo applications of this new natural medication are strongly suggested.

## Figures and Tables

**Table 1:** Minimum inhibitory concentrations of *Punica granatum* extracts.

Bacteria	Extracts	Samples (Provinces)		
		Isparta	Denizli	Mugla
		MIC Values (µg/mL)		
*S. aureus*-17	EE	13,000	13,000	13,000
	ME	6500	13,000	13,000
	WE	13,000	nt	6500
*S. aureus*-18	EE	a	13,000	13,000
	ME	13,000	13,000	6500
	WE	a	nt	6500
CNS-22	EE	a	a	13,000
	ME	13,000	13,000	13,000
	WE	a	13,000	13,000
CNS-29	EE	13,000	13,000	13,000
	ME	13,000	13,000	6500
	WE	a	a	13,000
CNS-32	EE	a	13,000	13,000
	ME	a	13,000	13,000
	WE	a	a	13,000
CNS-33	EE	a	13,000	6500
	ME	13,000	13,000	6500
	WE	a	a	13,000
CNS-36	EE	a	6500	13,000
	ME	a	a	13,000
	WE	a	a	13,000
CNS-37	EE	a	13,000	13,000
	ME	a	13,000	a
	WE	a	a	a

*S. aureus*: *Staphylococcus aureus*; CNS: coagulase-negative *Staphylococcus*; EE: ethanol extract; ME: methanol extract; WE: water extract; nt: not tested; a: MIC could not be determined at the concentrations tried.

## Data Availability

The data presented in this study are available upon reasonable request from the corresponding author.
